# A Role for Naturally Occurring Alleles of Endoplasmic Reticulum Aminopeptidases in Tumor Immunity and Cancer Pre-Disposition

**DOI:** 10.3389/fonc.2014.00363

**Published:** 2014-12-19

**Authors:** Efstratios Stratikos, Athanasios Stamogiannos, Efthalia Zervoudi, Doriana Fruci

**Affiliations:** ^1^National Center for Scientific Research Demokritos, Athens, Greece; ^2^Department of Paediatric Haematology/Oncology, IRCCS, Ospedale Pediatrico Bambino Gesù, Rome, Italy

**Keywords:** aminopeptidase, antigen presentation, cancer, SNP, cytotoxic responses, adaptive immunity, innate immunity, polymorphism

## Abstract

Endoplasmic reticulum aminopeptidase 1 and 2 (ERAP1 and ERAP2) are key components on the pathway that generates antigenic epitopes for presentation to cytotoxic T-lymphocytes (CTLs). Coding single nucleotide polymorphisms (SNPs) in these enzymes have been associated with pre-disposition to several major human diseases including inflammatory diseases with autoimmune etiology, viral infections, and virally induced cancer. The function of these enzymes has been demonstrated to affect CTL and natural killer cell responses toward healthy and malignant cells as well as the production of inflammatory cytokines. Recent studies have demonstrated that SNPs in ERAP1 and ERAP2 can affect their ability to generate or destroy antigenic epitopes and define the immunopeptidome. In this review, we examine the potential role of these enzymes and their polymorphic states on the generation of cytotoxic responses toward malignantly transformed cells. Given the current state-of-the-art, it is possible that polymorphic variation in these enzymes may contribute to the individual’s pre-disposition to cancer through altered generation or destruction of tumor antigens that can facilitate tumor immune evasion.

## Part I: ERAP1/2 and Cancer

### Mechanisms used by the immune system to fight cancer

The immune system plays a dual role in cancer, on one hand suppressing tumor growth by eliminating cancer cells or inhibiting their outgrowth, and on the other hand promoting tumor progression by selecting tumor cells able to survive inside an immunocompetent host (1–7). The dynamic process that integrates these opposing functions of the immune system – host protection and tumor progression – is termed “cancer immunoediting” and consists of three phases: (i) elimination, (ii) equilibrium, and (iii) escape.

In the elimination phase, cells of innate and adaptive immunity work together to identify and destroy nascent cancer cells, before they become clinically apparent tumors. If a cancerous cell variant is not eliminated in this phase, it may enter the equilibrium phase and remain in a state of functional dormancy under control of cells and mediators of the adaptive immunity. In this phase, the immune system controls tumor growth but does not eliminate it. Cancer cells that acquire mutations can become: (i) invisible to adaptive immunity, i.e., antigen-loss variants or tumor cells that develop defects in antigen-processing or presentation, (ii) insensitive to immune effector mechanisms, or (iii) induce an immunosuppressive state within the tumor microenvironment, enter the escape phase and grow progressively eventually becoming visible tumors. The achievement of the last phase is indicative of a failure in the adaptive immune cells to provide protection from tumor development due to selection of poorly immunogenic tumor cell variants during the equilibrium phase.

Many different cells belonging to the innate and adaptive immunity play an active role in cancer control, from the earliest stages of transformation to the terminal phase of widespread metastasis. The immune cells most involved in the host protection from development of cancer are natural killer (NK) cells and cytotoxic T-lymphocytes (CTLs).

NK cells provide the first immune defense against infections and tumor transformation through recognition and killing of aberrant cells. Their function is finely tuned by the interaction of activating and inhibitory receptors with their specific ligands expressed on target cells (8). Activating receptors recognize ligands expressed on stressed, infected, or transformed cells, whereas inhibitory receptors, prevent NK cell activation upon interaction with major histocompatibility complex (MHC) class I molecules expressed on target cells (9–14). The reduced surface expression of MHC class I molecules and acquisition of activating ligands in virally infected and transformed cells make these cells particularly vulnerable to NK cell-mediated killing.

CTLs constantly monitor peptide-MHC (pMHC) class I complexes on the cell surface and eliminate virally infected or transformed cells expressing novel peptides derived from abnormal gene products. The generation of these peptides is central in the regulation of CTL and NK cell responses against altered cells. Aberrant antigenic peptide generation can lead to either immune evasion or to immune responses against normal cells, initiating or sustaining autoimmune reactions. Antigenic peptides are generated by the concerted action of multiple components of a biochemical pathway termed MHC class I antigen-processing and presentation.

### Pathway of antigen-processing and presentation

Antigen generation generally starts with the ubiquitin–proteasome pathway where proteins are tagged for proteolytic destruction by the proteasome although other cytosolic peptidases often play important roles (15). The immune cell variant of the proteasome, called immunoproteasome, is more efficient in generating longer peptides, which are often N-terminally extended, compared to the final antigenic epitopes (16). A fraction of these precursors are transported by a specialized transporter [Transporter Associated with antigen-Processing (TAP)] into the endoplasmic reticulum (ER) for further processing. Inside the ER, the precursors are further trimmed by ER-resident aminopeptidases, ER aminopeptidase 1 (ERAP1) and ER aminopeptidase 2 (ERAP2), which excise the N-terminal extensions generating mature antigenic peptides (17, 18). These peptides then bind onto nascent MHC class I molecules with the help of a multi-protein complex termed the peptide loading complex. The pMHC class I complexes are then translocated to the cell surface where they can interact with specialized immune system (19).

### Role of ERAP1 and ERAP2 in the generation of antigenic epitopes

The key role of ERAP1 and ERAP2 in the generation of antigenic epitopes has been repeatedly demonstrated in several cell lines and mouse models. Reduction of ERAP1 expression by RNA interference results in drastically defective presentation for some antigenic epitopes (17, 18, 20, 21) and enhanced presentation or no effect for others. Based on these studies, it has become apparent that ERAP1 has a complex, multifaceted role on the generation of the immunopeptidome (22–24).

In mice models, the complete loss of ERAAP expression (the mouse homolog of ERAP1) specifically inhibited surface expression of MHC class I, but did not affect the expression of MHC class II molecules (17). The expression of pMHC class I on the cell surface depends on the quantity as well as the quality of the peptide supply. In ERAAP-deficient cells, the reduction of MHC class I molecules was due to faster dissociation of pMHC class I from the cell surface rather than to a slower rate of pMHC class I assemble in ER. As a result, ERAAP is important for generating optimal peptides that yield stable pMHC class I complexes (25). Moreover, Hammer et al. observed that the absence of ERAAP disrupts the pMHC class I repertoire in professional and non-professional antigen-presenting cells. From *in vivo* studies emerged that ERAP1 plays an important role in immune response to viruses, either enhancing or reducing CTL responses to particular viral epitopes and, thereby, helping establish immunodominance hierarchies (25). Undauntedly, expression of endogenous pMHC class I is essential for the generation and maintenance of the normal CD8^+^ T cell responses. Splenocytes from ERAAP-deficient mice display an alternative repertoire of peptides as well as differences in the stability of pMHC class I molecules characterized from a diminished ability to elicit HY-specific CD8^+^ T cell responses. Interestingly, immunization of ERAAP-deficient mice with splenocytes from wild-type mice resulted in potent CD8^+^ T cell responses, suggesting that ERAP1 plays an important role in modifying antigenic peptides and, paradoxically, its absence enhances immunogenicity (25, 26).

ERAP2, the second aminopeptidase demonstrated to be involved in antigen trimming in the ER, is highly homologous to ERAP1 but has distinct specificity (27–29). ERAP1 and ERAP2 have been suggested to perform antigenic peptide trimming in a coordinated manner by forming a functional heterodimer (18, 30). Saveanu et al. performed RNA interference to examine the roles of ERAP1 and ERAP2 in trimming of various precursors of the model HIV env epitope, using two different cell lines. The effect of ERAP2 knockdown on cell-surface MHC class I expression and epitope presentation was similar to that of ERAP1 knockdown, suggesting equivalent functions of the two enzymes in the cells studied. Also the greater effect of the double knockdown in some cases suggests that each enzyme can function independently, so that their effects are additive (18).

Overall, the exact effect of the ERAP1 and ERAP2 activities on antigen presentation can be highly variable and difficult to predict. Any factor that can influence the generation of the immunopeptidome may contribute to this, including the cell line used, the MHC class I alleles, whether the cell contains immunoproteasomes or constitutive proteasomes, the activities of cytosolic aminopeptidases and the sequence of the epitope studied. Regardless, ERAP1 and ERAP2 are undoubtedly important factors that influence the generation of the immunopeptidome, with ERAP1 having a dominant role (18, 22, 25).

### ERAP1 in innate immunity

ERAP1 has been found to play important roles in innate immune responses. Namely, ERAP1 has been involved in the shedding of cytokine receptors including the type I TNF receptor (TNFR1), type I IL-6 receptor (IL-6Ra), and type II IL-II decoy receptor (31–33). Additionally, macrophages were found to produce a secreted form of ERAP1 in response to interferon-γ and liposaccharides through a TLR-mediated mechanism that leads to enhanced phagocytosis (34, 35). Similarly, human PBMCs exposed to ERAP1 externally are activated and show enhanced production of cytokines and chemokines, through mechanisms involving the NLRP3 inflammasome (Aldhamen et al. *J Innate Immunity*, in press). This secreted form of ERAP1 was found to be enzymatically identical to the ER-retained form and only differ in glycosylation patterns consistent with maturation through the secretory pathway. Since ERAP1 does not contain an ER retention signal it has been hypothesized that it is normally retained inside the ER through interactions with specific ER-resident proteins and can be secreted when these interactions are saturated or disrupted (34).

The function of ERAP1 in regulating antigen presentation can also lead to altered NK cell immune responses. ERAP1 knock-out mice exhibit exaggerated innate immune responses early during pathogen recognition and show increased activation of NK and NKT cells and production of inflammatory cytokines (36). ERAP1 silencing in T cell lymphoma RMA results in tumor rejection in syngeneic mice by triggering NK cells and subsequently T cell (CD4^+^ and CD8^+^) anti-tumor responses. This rejection does not depend on a simple quantitative reduction in surface MHC class I expression, but is rather the result of changes in the MHC class I-peptide repertoire, because replacement of endogenous peptides with high-affinity mature antigenic peptides was sufficient to rescue the inhibitory activity of NK cells (37). Furthermore, ERAP1 knock-out mice show high frequencies of terminally matured as well as licensed NK cells expressing Ly49C and Ly49I receptors consistent with enhanced NK activation by pro-inflammatory stimuli in those mice (36). Together, these findings suggest an important role for ERAP1 in modulating innate immune responses during the earliest stages of pathogen recognition, a role that may be of particular importance to immune responses toward malignantly transformed cells.

### Altered levels of ERAP1 and ERAP2 can facilitate tumor immune evasion

The enzymatic activity and expression levels of the mouse homolog of ERAP1, ERAAP, have been demonstrated to be key for the immune evasion of tumor cells in two distinct murine models. In one study, the authors showed that ERAP1 down-regulation was sufficient to stimulate the cytotoxic activity of NK cells and to result in tumor growth arrest (37). In another study, down-regulation of ERAP1 elicited specific CTL responses against a cryptic tumor-associated antigen that was normally destroyed by ERAP1, resulting in tumor growth arrest and enhanced survival (38). These two studies clearly demonstrated that ERAP1 expression can be critical for immune evasion of solid tumors. Moreover, by using cell lines with two different levels of ERAAP expression levels James et al. established that the induction of anti-tumor immune responses can be titrated based on ERAAP activity, laying the groundwork for the hypothesis described here (38).

In humans, defective expression of components of the antigen-processing machinery has been associated with the progression and clinical outcome in several types of cancer. The availability of specific ERAP1 and ERAP2 antibodies has allowed researchers to investigate the expression and tissue distribution of these enzymes in a large number of tumor cells of various origins.

In one study, expression of ERAP1 and ERAP2 was detected in all tumor cell lines examined, including melanoma and various type of carcinomas, although at highly variable levels and independently of each other (39). The amount of ERAP1 appears to be more closely coordinated with cell-surface HLA class I molecules, suggesting a secondary involvement of ERAP2 in the generation of ligands for HLA class I molecules at least on quantitative levels. However, this study did not consider that ERAP2 is missing in 25% of the population (40). Notably, upregulation of ERAP1 and ERAP2 in ERAP-low tumor cells was found to enhance HLA class I surface expression, suggesting that abnormal HLA class I levels in tumor cells may result from defective expression of these enzymes (39).

In another study, a heterogeneous expression of ERAP1 and ERAP2 was detected in a panel of 28 melanoma cell lines (41). A concordant expression between mRNA and proteins for these genes was detected in many cell lines, except four in which an aberrant ERAP2 transcript resulted in total absence of ERAP2 protein expression.

Expression of ERAP1 and ERAP2 was subsequently investigated in 39 different cell types derived from 24 normal non-lymphoid tissues and their malignant counterparts (42). In normal tissues, expression of ERAP1 and ERAP2 was limited to epithelial components. The two enzymes were co-expressed, singly expressed or not expressed, depending on the cell type. HLA class I expression appeared to be independent of ERAP1 and ERAP2 expression and only in nine cell types it was coordinated with both enzymes. In tumor samples, the expression of either or both enzymes was retained, lost, or acquired as compared to the normal counterparts, depending on the tumor histotype. Loss of at least one enzyme was the most frequent phenotype accounting for 86% tumors. Remarkably, in four types of carcinomas (breast, ovary, liver, and lung carcinomas) arising from normal counterparts co-expressing ERAP1 and ERAP2, none of the 26 tested samples retained this phenotype. ERAP1 was lost in all tested breast, ovary, and lung carcinomas and in 6 out of 7 liver carcinoma samples tested, whereas ERAP2 was retained only in 9 out of 26 samples. The double-negative phenotype was significantly associated with lack of detectable HLA class I molecules. Thus, *in vivo* transformation affects the expression of ERAP1 and ERAP2, together and individually, leading to losses, gains, or imbalances.

In another study, expression of ERAP1 and ERAP2 was investigated in 300 normal kidney tissues and 334 renal cell carcinoma lesions (43). A heterogeneous and discordant expression of the two enzymes was detected in the different regions of the normal kidney epithelium. In renal cell carcinomas, ERAP1 and ERAP2 appear to have a different behavior, being the first more frequently up-regulated and the latter more frequently down-regulated, as compared to the normal counterpart. None of the clinical parameters investigated was found to be associated with ERAP1 and ERAP2 expression.

ERAP1 expression was also tested in 101 cervical carcinoma patients including adenocarcinomas and squamous cell carcinomas, and correlated with clinical outcome (44). ERAP1 expression was observed in most cases (85 out of 101) and overall never totally lost. Partial ERAP1 loss was significantly associated with reduced overall survival and disease free survival. In multivariate analysis, ERAP1 down-regulation was demonstrated to be an independent predictor for worse overall survival and disease free survival, and significantly associated with lymph node metastases.

In another study, ERAP1 expression was examined in 50 esophageal carcinoma lesions and compared with clinico-pathological parameters (45). In these tissues, ERAP1 expression was lost or down-regulated in 20 and 28% of cases, respectively, and significantly associated with the depth of tumor invasion. The authors showed that ERAP1 expression was partially or totally lost in cervical intraepithelial neoplasia and cervical squamous cell carcinoma as compared to normal epithelium of uterine cervix (46), but association with clinical outcome was not investigated.

Taken together, these studies suggest that normal function of ERAP1 and ERAP2 is required for NK cell- and T cell-mediated anti-tumor immunity. As a result, the deregulated expression of these enzymes found in tumors may cause improper antigen-processing and contribute to escape for host immune surveillance.

## Part II: ERAP1/2 SNPs

### ERAP1/2 SNPs and association to human disease

**Figure 1:**
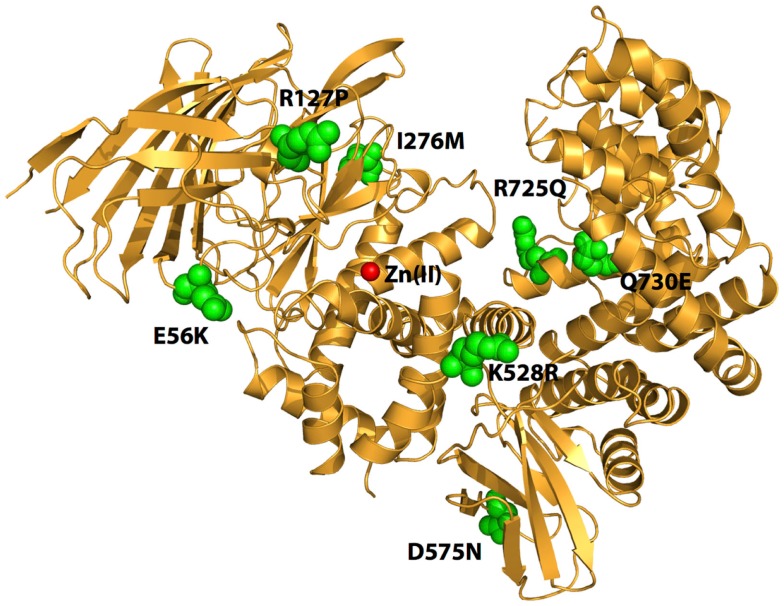
**Schematic representation of the crystal structure of ERAP1 (PDB code: 3MDJ)**. The amino acids at the sites of polymorphic variation that have been found to either affect enzyme function or to associate with pre-disposition to cancer are indicated by green spheres. The catalytic Zn(II) atom at active site of the enzyme is shown by a red sphere. Note that the polymorphic sites are distal to the active site and distributed throughout the enzyme.

Both ERAP1 and ERAP2 are naturally polymorphic and more than a dozen coding single nucleotide polymorphisms (SNPs) in their genes have been associated with pre-disposition to a variety of human diseases primarily of autoimmune etiology (Figure 1; Table 1). Such links are more evident for diseases that are strongly associated with particular HLA class I alleles, implying that the role of ERAP1 and ERAP2 in disease pathogenesis is through the pathway of antigen-processing and presentation. The most prominent example is the association of ERAP1 with Ankylosing Spondylitis (AS), a chronic inflammatory rheumatopathy of the lower spine with an autoimmune etiology. GWAS studies have consistently shown that various ERAP1 coding SNPs are associated with pre-disposition to AS (47–49). The variants that appear to confer risk susceptibility are: rs17482078-C (R725Q), rs30187-T (K528R), rs2287987-T (M349V), rs26653-C (R127P), rs10050860-C (D575N), and rs27044-G (Q730E), with each individual one contributing odd ratios of about 1.3–1.4 (47, 48, 50). Due to the strong linkage disequilibrium in the ERAP1/2 locus, specific haplotypes have been associated with either pre-disposition or protection against AS. A pre-disposing haplotype has been proposed to comprise of 730E, 575D, and 528K and a protective one of 528R, 276I, and 127P (51). Another pre-disposing haplotype also carries an ERAP2 SNP (ERAP1 730Q, ERAP1 528K, and ERAP2 392N) (52). Evans et al. showed that ERAP1 contribution to AS could be primarily attributed to rs30187-T (528K), with secondary effects found for rs10050860-C (575D) and rs17482078-C (725R) (48). Most notably ERAP1 is associated with AS only in the presence of at least one HLA-B27 allele, which is also the greatest known AS risk factor (48, 49) suggesting that the pathogenic effect is mediated through aberrant generation of HLA-B27 ligands either in the context of arthritogenic self-peptides or the generation of pro-inflammatory non-canonical HLA-B27 structures.

**Table 1:** **Most common ERAP1 and ERAP2 SNPs, relation to disease, HLA, and functional consequences**.

	ERAP1/2 SNP	Disease association	HLA class I link	Affects
ERAP1	rs3734016 (E56K)	HPV-induced cervical carcinoma		Expression levels
	rs26653 (R127P)	Ankylosing spondylitis, HPV-induced cervical carcinoma		Expression levels
	rs2287987 (M349V)	Ankylosing spondylitis		
	rs30187 (K528R)	Ankylosing spondylitis, psoriasis, essential hypertension, multiple sclerosis	B*27 Cw*0602	Activity and specificity
	rs10050860 (D575N)	Ankylosing spondylitis, Behçet disease		Activity
	rs17482078 (R725Q)	Ankylosing Spondylitis, Behçet disease	B*51	Activity
	rs27044 (Q730E)	Ankylosing spondylitis, HPV-induced cervical carcinoma		Activity and specificity
ERAP2	rs2549782 (K392N)	Ankylosing spondylitis, preeclampsia, resistance to HIV infection		Activity and specificity
	rs2248374 (non-coding)	Ankylosing spondylitis		Expression levels

The association of ERAP1 with diseases in the context of specific HLA class I alleles is also observed in the cases of Behçet disease (BD) and Psoriasis. A GWAS study in a Turkish population, where BD is frequently encountered, identified rs10050860-T (575N) and rs17482078-T (725Q) as increased risk variants, with odd ratios significantly higher for HLA-B51 positive individuals (53). Similarly to HLA-B27 and its association to AS, HLA-B51 is the greatest known risk factor for BD. A recent study showed that six positions within the peptide-binding cavity of MHC-I molecules are primarily responsible for the association of particular HLA alleles (including HLA-B51) with BD, implying that aberrant peptide binding by HLA-B51 is central to the pathogenesis of BD (54). Another GWAS study using a population of European ancestry demonstrated that rs30187-T (528K) increases risk for psoriasis, but only in the presence of at least one copy of the allele HLA-Cw*0602 (55). In addition to these well-established cases, ERAP1 SNPs, with rs30187 as a prominent example, have also been associated with other diseases. Specifically, the polymorphism rs30187-C (528R) has been associated with essential hypertension (56) and the rs30187-T (528K) variant with Multiple Sclerosis (57).

ERAP2 has been associated with AS independently of ERAP1. Two functional SNPs have been described: coding rs2549782 (K392N) and rs2248374, a SNP that greatly influences ERAP2 expression (49). Furthermore, another ERAP2 SNP (rs2910686) was associated with AS in HLA-B27-negative cases (49). ERAP1 and ERAP2 SNPs can form haplotypes that may be more relevant to disease pathogenesis (52). The ERAP2 coding SNP rs2549782 (K392N) is also linked with preeclampsia and with resistance to HIV-1 infection (58–61). The minor allele G is significantly associated with preeclampsia in African-American and Australian populations, but not in a Chilean population (58, 60, 61). A case-control study in a cohort of Italian HIV-exposed seronegative individuals showed that the homozygote GG genotype (encoding Lys/Lys) is overrepresented in the HIV-exposed seronegative sample (59). Recently, Kuiper et al. identified variants near the ERAP2 locus that are associated with birdshot chorioretinopathy (BCR) (62). This GWAS study identified rs7705093-T (OR: 2.3), to be in perfect linkage disequilibrium with rs10044354, a variant that affects ERAP2 protein expression levels, with individuals homozygous for the C allele showing almost none ERAP2 expression. BCR is a useful prototype of disease-HLA association since it exhibits the strongest documented HLA class I association for a human disease (>95% of cases carry the HLA-A29 allele) implicating antigenic peptide processing and presentation in the pathogenesis of this disease.

Overall, a very strong genetic link between ERAP1/2 SNPs and HLA-associated autoimmunity has been established and has contributed to our understanding of the pathogenesis of these diseases. The genetic variability of ERAP1 and ERAP2 appears to constitute part of the natural variability of immune responses and may therefore contribute to pre-disposition to any number of diseases actively fought by the adaptive immune response.

### Polymorphic state of ERAP1/2 affects enzyme function and antigen generation

The multitude of genetic and population studies linking ERAP1 and ERAP2 SNPs to pre-disposition to autoimmunity and viral infections prompted several research groups to examine the effects of the identified polymorphic variations on the enzyme’s biological function and molecular mechanism. To date, most studies have focused on the most well disease-associated SNPs, namely K528R, D575N, R725Q, and Q730E.

The SNP at position 528 has been repeatedly demonstrated to influence ERAP1 enzymatic activity. Various *in vitro* studies have shown that the 528R variant exhibits less enzymatic activity, compared to the 528K (48, 63–66). Cell-based experiments have also demonstrated the importance of that position: HeLa cells transfected with ERAP1 528R and a HLA-B27 peptide precursor displayed a reduced amount of HLA-B27 molecules on the cell surface compared to ERAP1 528K transfectants (64). Furthermore, the presentation of specific MHC class I epitopes was negatively influenced in cells transfected with the 528R variant and their N-extended precursors, as shown by CTL activation assays (67, 68).

Studies regarding polymorphic positions Q730E, D575N, and R725Q have been more complicated to interpret. *In vitro* assays have suggested that position 730 may or may not have an influence in the enzymatic activity, depending on the substrate used (63, 64). Regardless, a cell-surface HLA class I expression assay showed that this SNP could influence the generation of a specific HLA-B27-restricted epitope (64). Some studies showed that position 575 does not influence ERAP1 activity *in vitro* (48, 63), while another study showed that the 575N variant exhibits greater activity compared to 575D (66). Regarding position 725, Evans et al., using recombinant enzymes, showed that 725Q negatively influences enzymatic activity toward a model fluorigenic peptide substrate (48), but an *in vitro* CTL activation assay did not find any influence of that position to the presentation of an HLA-B27 epitope (68). The complex landscape of functional effects found for these SNPs may be attributed to differences in substrates or assays used, or to differences in background SNPs that are not always consistent between studies. Indeed, in several studies the effects of ERAP1 SNPs have been found to be strongly epitope dependent and to include effects on other mechanistic aspects of ERAP1 peptide trimming, such as substrate inhibition and product activation phenomena (64, 66, 69).

SNPs in ERAP1 and ERAP2 are often co-inherited as complex haplotypes and may have strong synergism with each other. Unfortunately, most functional studies until now have focused on analyzing the effects of single SNPs and as a result the functional effects of particular disease-associated ERAP1/ERAP2 haplotypes are not always clear especially since the synergisms between individual SNPs are not known. Recently, Seregin et al. analyzed two ERAP1 haplotypes of five SNPs for their effects on HLA-B27 restricted presentation in relation to the pathogenesis of AS and concluded that the high-risk haplotype resulted in reduction in presentation of multiple antigens (70). In another study, Reeves et al. identified nine separate naturally occurring haplotypes in a small population sample based on the five most disease-related SNPs (positions: M349V, K528R, D575N, R725Q, and Q730E) (67). They evaluated the trimming activity of these alleles using a CTL activation assay and showed that each allele exhibits a different activity that is not only dependent on the allele itself but also on the N-terminal extension of the peptide. This study was recently extended to AS patients, showing that ERAP1 haplotypes were clearly stratified in individuals with AS compared to healthy controls and that these functional alleles were poor in generating optimal peptide ligands for HLA-B*2705 (71). A study focusing on the combined effects of positions 528 and 575 showed that the latter position was dominant in determining enzyme activity (66). Recently, Chen et al. demonstrated that the polymorphic variation in position 730 is critical for rescuing the reduced CTL activation found in the presence of the 528R SNP (68). Overall, ample evidence for strong synergism amongst ERAP1 SNPs are available, although we have very little insight on how these SNPs that are scattered all throughout the ERAP1 structure can cooperate to affect the activity of the enzyme.

The influence of ERAP1 polymorphic context has also been studied in the context of the HLA-B27 restricted immunopeptidome and the pathogenesis of AS. Garcia-Medel et al. showed that the presence of ERAP1 with all the AS-pre-disposing polymorphisms (349M, 528K, 575D, 725R, and 730Q) ensured efficient peptide trimming and a higher HLA-B27 stability, compared to the ERAP1 with the AS-protective SNPs (349V, 528R, 575N, 725Q, and 730E) (72). Extending this work, Martin-Esteban et al. showed that synergism between SNPs at positions 528 and 575 can have important effects on cell-surface HLA-B27 presented ligands and more recently Garcia-Medel et al. showed that the particular B27 subtype is critical in determining these effects (66, 73). It appears that the interplay between ERAP1 SNPs and HLA class I subtypes is the key to determining the cell-surface immunopeptidome and concomitant cytotoxic responses.

Two ERAP2 SNPs have been shown to have specific effects on the enzyme’s biological function: the coding polymorphism rs2549782 (N392K) and the non-coding rs2248374. ERAP2 polymorphism N392K alters the activity and specificity of ERAP2 in a manner much more pronounced compared to the effects described for all coding SNPs of ERAP1 (74). Specifically, *in vitro* cell-based analysis showed that the 392N variant is much more effective in trimming hydrophobic N-terminal residues from antigenic peptide precursors compared to 392K. In contrast, the rs2248374-G allele can determine ERAP2 expression levels by inducing mRNA instability and non-sense mediated decay (40). The biological consequence of that frequently encountered allele (about 0.5 frequency in six different populations studied) has also been demonstrated; rs2248374-G homozygotes produce no detectable ERAP2 and have reduced levels of MHC class I expression on B-cell surfaces.

Similarly to ERAP1, these ERAP2 SNPs often organize to distinct haplotypes. SNP rs2248374 is in linkage disequilibrium with rs2549782 in various populations studied, thus effectively allowing only the expression of 392K variant (40). A noteworthy exception is the Chilean population where the two SNPs are not in linkage disequilibrium, allowing the expression of 392N variant (61). Nevertheless, no Chilean genotype was found homozygous for the 392N allele, implying a possible negative selection for individuals that are homozygous for that allele. Interestingly, the recent association of ERAP2 expression with pre-disposition to the inflammatory autoimmune disease BCR solidified the idea that ERAP2 polymorphic variation and its effects on antigen-processing may have important repercussions on adaptive immune responses (62).

### ERAP1 SNPs and pre-disposition to virally induced cancer

Genetic variations in genes encoding components of the antigen-processing pathway have been associated with risk of occurrence malignancy and in particular with cervical carcinoma. Mehta and colleagues analyzed the effect of different ERAP1 SNPs and haplotype combinations on the risk of developing human papillomavirus (HPV)-induced cervical carcinoma.

In the first study, the authors identified two common ERAP1 polymorphisms, R127P and Q730E, significantly associated with increased risk of cervical cancer (75). A haplotype combination consisting of four SNPs, including the minor alleles at R127P and Q730E loci and a major allele at the TAP1 R651C and LMP7 Q145K loci, was significantly associated with a threefold increased cervical carcinoma risk (75). The authors estimated that 12% of all cervical carcinoma cases were attributable to the occurrence of this haplotype combination (75).

In a subsequent study, the same group investigated which genetic ERAP1 variation affected tumor progression and overall survival in cervical carcinoma patients, and provided the first indication of association of ERAP1 SNPs with ERAP1 protein expression (76). Genotype distributions at the R127P, I276M and K528R were significantly associated with the presence of lymph node metastases. Heterozygosity of E56K and minor allele homozygosity at the R127P loci were significantly associated with decreased overall survival. Multivariate analysis performed on E56K and R127P genotypes combined with prognostic factors, revealed that the two SNPs loci were not independent predictors of survival. The R127P variant and the E56K–R127P haplotype were significantly associated with ERAP1 protein expression, with heterozygosity of both individual SNPs and the haplotype consisting of a major allele at the E56K locus and a minor allele at the R127P, being significantly associated with normal ERAP1 expression and better overall survival. Heterozygosity for E56K–R127P haplotype was found to be an independent predictor for overall survival and lymph node metastasis.

The association between ERAP1 SNPs and cervical carcinoma risk or patient survival may be explained by different mechanisms, all related to altered ERAP1 function. Genetic variations at these individual SNPs have been found to affect the expression and stability of transcripts and proteins, to reduce trimming activity or modify substrate specificity (48, 64, 76). All these effects may affect the ability of HPV to establish persistent infections, but also the ability of transformed cells to evade immune surveillance. The information gathered by these studies has helped to establish a new paradigm on how the polymorphic variation of the adaptive immune system can play a role on both cancer development and prognosis. Although the importance of viral control in the pre-disposition to virally induced cancer is easy to understand, this work has opened the possibility that variable immune responses in the population may also play important roles to cancer pre-disposition.

### ERAP1 and ERAP2 SNPs and pre-disposition to cancer – a hypothesis

Current knowledge on the roles of ERAP1 and ERAP2 in the human immune response have gradually led to the maturation of the hypothesis that the naturally occurring polymorphic variation in ERAP1 and ERAP2 may play significant roles in the pre-disposition to certain cancers, as well as their progression and prognosis. More specifically, the rational behind this hypothesis is based on the following points:

1. CTL and NK cell responses against malignantly transformed cells are important for eradicating tumor cells often at the early stages of carcinogenesis.
2. Establishment of solid tumors often requires adaptive measures from the transformed cells that lead to the evasion of immune responses. Such adaptive measures include changes in antigen-processing and presentation in order to suppress the presentation of tumor-specific antigenic epitopes while also evading NK-cell recognition.
3. Polymorphic variation in ERAP1 and ERAP2, alters their function and their ability to generate antigenic peptides and controls cytotoxic reactions against antigen-presenting cells. This is now well established in autoimmunity and viral infections.
4. ERAP1 and ERAP2 expression levels, which undoubtedly affect effective enzyme activity, are consistently altered in tumors, presumably as an adaptive measure for immune evasion.
5. ERAP1 function can regulate innate immune responses and the production of inflammatory cytokines, a function that can either help eradicate cancer cells or contribute to localize inflammation that can promote tumor growth.

**Figure 2:**
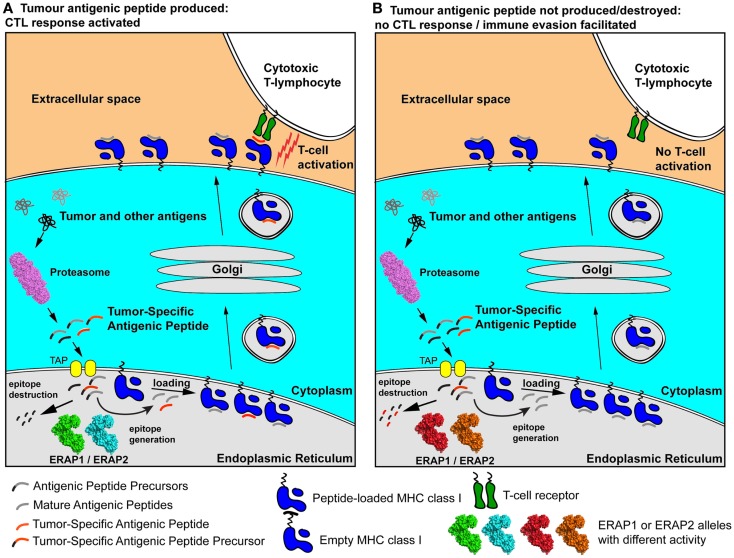
**Schematic representation of two extreme scenarios leading to either effective cytotoxic responses against a cancer cell (A) or immune evasion (B)**. A tumor-specific antigenic epitope or its N-terminal extended precursor is generated by the proteasome and transported into the ER. There, it is further processed by ERAP1/ERAP2 alleles. In case A, the “green” and “cyan” allele effectively generate the mature epitope (red line), which is then loaded onto nascent empty MHC class I and presented to the cell-surface, activating antigen-specific T cells. In case B, hyper-active or hypo-active ERAP1/ERAP2 alleles (in red and orange) either fail to produce the mature epitope or destroy it by generating peptides too small to bind onto MHC class I molecules. As a result, the epitope is not presented on the cell surface and no T cell activation occurs, facilitating immune evasion. The allelic state of ERAP1 and ERAP2 can therefore influence anti-tumor adaptive immune responses pre-disposing individuals to certain cancers by facilitating immune evasion at early stages of malignant development.

As a result, the polymorphic variability in ERAP1 and ERAP2 may affect the severity of early cytotoxic responses toward transformed cells and influence their chances to accrue genetic adaptations that will allow them to evade the immune system (Figure 2). Individuals carrying particular ERAP haplotypes in combination with specific HLA class I alleles may therefore be more prone to carcinogenesis, not because of facilitated malignant transformation but because they have an immune system that is less effective in mounting strong cytotoxic responses against cancer cells. Additionally, the function of ERAP1 in regulating inflammatory cytokine production may contribute to these effects by influencing the inflammatory state of the tumor microenvironment.

### Controlling ERAP1 and ERAP2 activity for treating cancer

In addition to the possible role of the activity of ERAP1 and ERAP2 on the normal immune control of cancer, a series of recent studies have highlighted that these two enzymes may be important pharmacological targets for boosting immune responses to established cancers. In one study, the genetic down-regulation of ERAP1 in cancer cells that establish solid tumors in mice resulted potent NK cell-mediated cytotoxic responses (37). In another study, the genetic down-regulation of ERAP1 in cancer cells resulted in strong CTL responses against a cryptic tumor antigen normally destroyed by over-active ERAP1 (38). In both studies, tumor growth was halted resulting in clear therapeutic outcome in mice. In the latter study, the effect was partially reproduced using the small molecular weight metallopeptidase inhibitor Leucinethiol (38). A recently developed potent ERAP1/ERAP2 inhibitor was successfully used to induce CTL responses against the cancer cells used in that study (77). Apart from the obvious extension of these studies conclusions to cancer immunotherapy, one additional conclusion may be drawn: inhibition of ERAP1 activity was not necessarily complete in either study, indicating that even moderate modulation of ERAP1 activity, such as seen in the normal polymorphic variation in this enzyme, may be sufficient to radically affect the potential of cancer immune surveillance.

### Concluding remarks – future research directions

In summary, we review the state-of-the-art on the role of ERAP1 and ERAP2 in adaptive and innate immune responses and their role on disease pathogenesis. Combination of knowledge on the role of polymorphic variation on those enzymes and disease pre-disposition with their role in cancer development has led to the formulation of a hypothesis on their direct role on cancer pre-disposition and prognosis. If this hypothesis holds to be true, haplotype analysis of components of the antigen-processing machinery may contribute to our understanding of cancer pre-disposition and complement other genetic findings on biochemical pathways that control malignant transformation. Because of the genetic heterogeneity of tumors however, it may be difficult to discern such effects at the tumor level and population studies may have to focus on the overall influence of this genetic locus on pre-disposition to specific cancers. Ongoing and future GWAS studies on cancer patients may yet reveal the importance of immune system variation to developing and fighting cancer, contributing to personalized treatments.

## Conflict of Interest Statement

The authors declare that the research was conducted in the absence of any commercial or financial relationships that could be construed as a potential conflict of interest.
